# Evolution by gene duplication of *Medicago truncatula PISTILLATA*-like transcription factors

**DOI:** 10.1093/jxb/erv571

**Published:** 2016-01-15

**Authors:** Edelín Roque, Mario A. Fares, Lynne Yenush, Mari Cruz Rochina, Jiangqi Wen, Kirankumar S. Mysore, Concepción Gómez-Mena, José Pío Beltrán, Luis A. Cañas

**Affiliations:** ^1^Instituto de Biología Molecular y Celular de Plantas Consejo Superior de Investigaciones Científicas & Universidad Politécnica de Valencia (CSIC-UPV), Ciudad Politécnica de la Innovación, Edf. 8E, C/ Ingeniero Fausto Elio s/n, E-46011 Valencia, Spain; ^2^Plant Biology Division, The Samuel Roberts Noble Foundation, 2510 Sam Noble Parkway, Ardmore, OK 73401,USA

**Keywords:** Duplicated B-function genes, functional analyses, MADS-box transcription factors, *Medicago truncatula*, molecular evolution, *PISTILLAT*A-like.

## Abstract

*PISTILLATA* (*PI*) is a member of the B-function MADS-box gene family, which controls the identity of both petals and stamens in *Arabidopsis thaliana*. In *Medicago truncatula* (*Mt*), there are two *PI*-like paralogs, known as *MtPI* and *MtNGL9*. These genes differ in their expression patterns, but it is not known whether their functions have also diverged. Describing the evolution of certain duplicated genes, such as transcription factors, remains a challenge owing to the complex expression patterns and functional divergence between the gene copies. Here, we report a number of functional studies, including analyses of gene expression, protein–protein interactions, and reverse genetic approaches designed to demonstrate the respective contributions of each *M. truncatula PI*-like paralog to the B-function in this species. Also, we have integrated molecular evolution approaches to determine the mode of evolution of *Mt PI*-like genes after duplication. Our results demonstrate that *MtPI* functions as a master regulator of B-function in *M. truncatula*, maintaining the overall ancestral function, while *MtNGL9* does not seem to have a role in this regard, suggesting that the pseudogenization could be the functional evolutionary fate for this gene. However, we provide evidence that purifying selection is the primary evolutionary force acting on this paralog, pinpointing the conservation of its biochemical function and, alternatively, the acquisition of a new role for this gene.

## Highlight

By studying the mode of evolution of *Medicago truncatula PISTILLATA*-like paralogs after duplication, we provide evidence of the complex dynamics underlying the evolution by gene duplication of the B-class MADS-box subfamily.

## Introduction

Transcription factors (TFs) involved in developmental innovations are a paradigmatic case of evolution by gene duplication, with most TF duplications being concomitant with the emergence of major morphological innovations in angiosperms (Purugganan *et al.*, 1995; Kramer *et al.*, 1998; Aoki *et al.*, 2004; Kim *et al.*, 2004). A particularly attractive model system suited to address the mechanisms of gene diversification in plant evolution is provided by the large family of MADS-box TFs (Theissen *et al.*, 2000; Airoldi and Davies, 2012).

Flower development is controlled by complex network interactions between TFs, most of them belonging to the MADS-box gene family (Schwarz-Sommer *et al.*, 1990; Wellmer *et al.*, 2014). These proteins form complexes that often interact with their own promoters and those of their targets to regulate their own and each other’s expression. They contain an N-terminal DNA-binding MADS domain, followed by the intervening (I) and keratin-like (K) regions, the latter being essential for dimerization and formation of high-order complexes (Yang *et al.*, 2003). Moreover, the highly variable C-terminal domain may have a role in protein complex formation and transcriptional regulation (reviewed by Kaufmann *et al.*, 2005).

B-function MADS-box genes are involved in the specification of petal and stamen identity in probably all angiosperms (Litt and Kramer, 2010). In *Arabidopsis thaliana*, this function is encoded by the combined activities of *PISTILLATA* (*PI*) and *APETALA3* (*AP3*) (Bowman *et al.*, 1989, 1991; Jack *et al.*, 1992). *PI* and *AP3* represent lineages that arose from a duplication event after the split between extant gymnosperms and angiosperms. Although several duplication events of the PI lineage have been documented throughout angiosperm evolution (Kramer *et al.*, 1998, 2003; Kramer and Irish, 2000; Kim *et al.*, 2004; Stellari *et al.*, 2004; Viaene *et al.*, 2009), few studies are available that combine functional and molecular analyses to understand the evolutionary forces that act on these duplicate gene lineages and the outcomes that ensure (Des Marais and Rausher, 2008).

Classic theory predicts that the genetic redundancy emerging from the duplication of genes relaxes the selection constraints on one gene copy allowing it to explore the genotype space and probe a wide range of phenotypes (Payne and Wagner, 2014). The evolutionary instability of genetic redundancy guarantees, however, the return of most duplicated genes to single-copy genes (Wolfe and Shields, 1997), which undermines the importance of the role of gene duplication in biological innovation. In contrast to this prediction, up to 30% of the genes in some organisms are duplicates (Blanc and Wolfe, 2004; Cui *et al.*, 2006), sparking the idea that a number of factors allow duplicated genes to persist, and thus innovate, within the genomes. Determining these factors remains a major goal of evolutionary biology. Expression and functional diversification of TFs are essential to allow the persistence in duplicate (Singh and Hannenhalli, 2008). The link between gene expression and the rate of evolution (Drummond *et al.*, 2005; Gout *et al.*, 2010), however plausible in TFs, remains a major unknown in the evolution of TFs after duplication.

Models have been proposed to explain how selection operates on duplicated genes (Innan and Kondrashov, 2010). Studies have identified many factors influencing the functional fates of duplicates, including selection for increased dosage (Conant and Wolfe, 2008), stoichiometric and gene balance (Birchler *et al.*, 2005; Freeling and Thomas, 2006), the position of genes in the protein interaction network (Alvarez-Ponce and Fares, 2012), and whether duplicates are the result of whole-genome, small-scale duplications or retropositions (Carretero-Paulet and Fares, 2012; Fares *et al.*, 2013; Keane *et al.*, 2014).

Arabidopsis homeotic floral genes are conserved in model legumes (Hecht *et al.*, 2005). *Medicago truncatula* contains four B-function MADS-box genes: two *AP3*-like (*MtNMH7* and *MtTM6*) (Roque *et al.*, 2013) and two *PI*-like paralogs (*MtPI* and *MtNGL9*) (Benlloch *et al.*, 2009), thus representing a good model system to study the effects of gene duplication and functional divergence within the B-function MADS-box lineages. Our previous functional characterization of the *Mt AP3*-like genes revealed that these paralogs have undergone a qualitative subfunctionalization process, concomitant with a complete partitioning of the expression pattern of the ancestral gene lineage (Roque *et al.*, 2013). Previous functional characterization of *MtPI*, one member of the duplicated *PI*-like genes, suggested that *MtPI* plays a major role in the specification of petal and stamen identity in *M. truncatula* (Benlloch *et al.*, 2009). However, the contribution of *MtNGL9* to floral development was unclear and its functional fate after gene duplication remains unknown. Here, we have integrated both functional analyses and molecular evolution studies to assess comprehensively the divergence in the B-function of duplicated *M. truncatula PI*-like genes and determine their mode of evolution after duplication.

## Materials and methods

### Plant material and growth conditions

*Medicago truncatula* cv. Jemalong lines R108 and A17, *Medicago sativa* cv. Regen SY-27, *Pisum sativum* cv. Alaska plants, and *Arabidopsis thaliana* cv. Landsber *erecta* (L*er*) plants were used in this study. Leguminous plants were grown in the greenhouse at 22 °C (day) and 18 °C (night) with a 16h light/8h dark photoperiod, in a mixture of soil/sand (3:1). Arabidopsis plants were grown in growth chambers at 21 °C under long-day (16h light) conditions, in a mixture of 1:1 perlite:vermiculite. Plants were irrigated with Hoagland No. 1 solution supplemented with oligoelements (Hewitt, 1966). The *mtpi-2* mutant allele was isolated in a previous screening (Cheng *et al.*, 2014).

### Isolation and sequence analysis of *PsNGL9* and *MsPI*

*PsNGL9* and *MsPI* coding regions were isolated to use their sequences in the molecular evolution approaches. *PsNGL9* (KJ470632) was obtained by reverse transcription–PCR (RT–PCR) from *Pisum sativum* floral cDNA samples using the primers MtNGL9-ATG/MtNGL9-638 (see Supplementary Table S4 at *JXB* online). *MsPI* (KJ470631) was obtained by RT–PCR from *M. sativa* RSY-27 floral cDNA samples using primers MtPI-ATG/MtPI-548 (see Supplementary Table S4). The *MsPI* 3'-untranslated region (UTR) and C-terminal region were obtained using the 3' rapid amplification of cDNA ends (RACE) system (Invitrogen) with nested gene-specific primer MsPI-363 (Supplementary Table S4). Sequence alignment and similarity comparisons of the coding region and inferred proteins were performed using CLUSTALW. Sequence alignments and similarity comparisons of several PI-like proteins were performed using the UNIPROT website (http://www.uniprot.org). MADS-domains (positions 1–61) were defined by the Prosite database (http://prosite.expasy.org/) using the AtPI protein sequence as the query.

### Phylogenetic tree

The evolutionary history was inferred by using the maximum likelihood method based on the JTT matrix-based model (Jones *et al.*, 1992). The tree with the highest log-likelihood value (–5459.065) is shown. Initial tree(s) for the heuristic search were obtained automatically by applying Neighbor-Joining and BioNJ algorithms to a matrix of pairwise distances estimated using the JTT model, and then selecting the topology with the superior log-likelihood value. A discrete Gamma distribution was used to model evolutionary rate differences among sites (5 categories +G, parameter=1.447). The rate variation model allowed for some sites to be evolutionarily invariable ([+I], 0.895% sites). The tree is drawn to scale, with branch lengths measured as the number of substitutions per site. The analysis involved 70 amino acid sequences obtained from GenBank, and two *PI*-like sequences that we have isolated from *M. sativa* and *P. sativum* (Supplementary Table S1). All positions containing gaps and missing data were eliminated. There were a total of 91 positions in the final data set. The substitution rates of the *PI*-like genes compared among different paralogs were inferred from the phylogenetic tree using the relative rate test implemented in MEGA 5 (Tamura *et al.*, 2011).

### RNA *in situ* hybridization

RNA *in situ* hybridization with digoxigenin-labeled probes was performed on 8 µm longitudinal paraffin sections of *M. truncatula* inflorescences as described previously (Ferrandiz *et al.*, 2000). A 298bp fragment of *MtPI* (504–801 from ATG) and a 243bp fragment of *MtNGL9* (511–753 from ATG) were introduced into the pGEM-T Easy vector. Digoxigenin-labeled RNA antisense and sense probes were synthesized by *in vitro* transcription using T7 and SP6 RNA polymerases, respectively. Signal was detected as a purple precipitate when viewed under the light microscope.

### Southern blot hybridization

Plant genomic DNA was extracted from leaves of *M. truncatula* cv. Jemalong, line A17 using standard procedures. A 10 μg aliquot of DNA was digested with *Eco*RI, *Bam*HI, and *Hin*dIII, and separated on a 0.7% agarose gel overnight. Southern blot hybridization was performed by the standard method using two different conditions (52 ºC and 65 ºC). cDNA probes were isolated by PCR using the primer pairs MtNGL9-511/MtNGL9-753 and MtPI-504/MtPI-801 for the *MtNGL9* and *MtPI* genes, respectively (see Supplementary Table S4).

### Yeast two-hybrid analysis

For construction of the two-hybrid plasmids, the cDNAs of the entire coding region of the *MtPI* and *MtNGL9* genes were subcloned into the pBTM116 two-hybrid vector (Vojtek *et al.*, 1993) to generate in-frame fusions with the LexA DNA-binding domain (BD), and the IKC fragments of *MtNMH7* and *MtTM6* genes were subcloned in pACT2 (Clontech) to generate in-frame fusions with the Gal4 transcriptional activation domain (AD). *Bam*HI/*Sal*I sites in the case of the pBTM116 constructs and *Bam*HI/*Eco*RI or *Bam*HI/*Xho*I sites in the case of the pACT2 constructs were added to primers used in the PCRs (see Supplementary Table S4). The yeast two-hybrid strain CTY10-5d was co-transformed with the appropriate constructs and transformants were selected on minimal medium (SD-Leu-Trp). Selection for interaction was performed on minimal medium (SD-Leu-Trp) containing X-Gal (80mg l^–1^) and 1× BU salts [Na_2_HPO_4_·7H_2_O (7g l^–1^); NaH_2_PO4 (3g l^–1^)]. Growth of yeast for blue staining was scored after 60h of incubation at 30 ºC (Fig. 4, bottom). Equivalent expression levels of MtPI and MtNGL9 LexA fusions were confirmed by western blot. In order to be able to quantify protein–protein interactions, we used a β-gal liquid assay with orthonitrophenyl-β-d-galactopyranoside (ONPG) as the substrate, measuring absorbance at 405nm. β-Gal activity was calculated essentially as described (Ludin *et al.*, 1998).

### Molecular characterization of the *Tnt1* insertion mutants *mtngl9-1* and *mtngl9-2*

The *M. truncatula* population used for the screening of mutants has been described in detail (Roque *et al.*, 2013; Cheng *et al.*, 2014; Serwatowska *et al.*, 2014). The *mtngl9* alleles were identified by PCR screening of a segregating population of ~10 000 independent lines, using primers annealing to the *MtNGL9* sequence (NGL9-F, Supplementary Table S4) in combination with primers annealing to the LTR borders of the *Tnt1* retroelement (Tnt1-R; Supplementary Table S4). PCR products (see Supplementary Fig. S3B) were obtained and cloned into the pGEM-T easy vector for sequencing. The *Tnt1* insertion in *mtngl9-1* is located at 360bp from the start codon, in the second exon (Supplementary Fig. S2A) and the *Tnt1* insertion in *mtngl9-2* is located at 183bp from the start codon, at the end of the first exon (Supplementary Fig. S2A). The R1 plants were genotyped using the following primer pairs: NGL9-F/Tnt1-R which amplified the T-DNA insertion, and NGL9-F/NGL9-590G or NGL9-F/NGL9-612G for the wild-type fragment in *mtngl9-1* and *mtngl9-2*, respectively (Supplementary Table S4; Supplementary Fig. S2B, C). Heterozygous lines NF14948.1 and NF14948.2 were self-pollinated. Approximately a quarter of the resultant progeny co-segregated with the *Tnt1* insertion. Gene expression of *MtNGL9* in the homozygous *Tnt1* mutants was performed by RT–PCR (Supplementary Table S4; Supplementary Fig. S2D).

### *Arabidopsis thaliana* transformation and genotyping

*MtPI* and *MtNGL9* cDNAs were cloned into the *SalI*/*Bam*HI sites of the pBINJIT60 vector (Guerineau and Mullineaux, 1993), a pBIN19 derivate (Clontech Laboratories). We used the primers MtPI-SalI/MtPI-BamHI for *MtPI* cloning, and MtNGL9-SalI/MtNGL9-BamHI for *MtNGL9* cloning (Supplementary Table S4). The transcription of *MtPI* and *MtNGL9* is under the control of a tandem repeat of the 35S promoter of *Cauliflower mosaic virus*. This vector was inserted into the *Agrobacterium tumefaciens* strain GV3101::pMP90(RK) and Arabidopsis plants were transformed according to standard procedures (Bechtold and Pelletier, 1998). For each construct, kanamycin-resistant lines were used for phenotypic and molecular characterization. Heterozygous transgenic lines *35S::MtPI* (4) and *35S::MtNGL9* (22) were used as the pollen donor for crosses to the homozygous mutant *pi-1* (Bowman *et al.*, 1989). The resulting progeny were allowed to self-fertilize, and plants containing the transgenes and homozygous for the *pi-1* allele (*pi-1/pi-1*;*35S::MtPI* or *pi-1/pi-1*;*35S::MtNGL9*) were identified in the next generation. To genotype the *pi-1* mutation, we designed a CAPS (cleaved amplified polymorphic sequences) marker using the primers CAPS-FOR/CAPS-REV (Supplementary Table S4) that amplify 729bp and the *Bse*GI enzyme thats cut only the wild-type PI sequence (Supplementary Fig. S6).

### RT–PCR analysis

Total RNA was isolated from floral apices from wild-type *M. truncatula* R108, *M. sativa* cv. RSY-27, and *P. sativum* cv. Alaska, and from *mtngl9* and *mtpi*-2 mutant flowers. Also, we isolated total RNA from leaves or floral buds of *35S::MtPI*, *35S::MtNGL9*, and *pi-1/pi-1*;*35S::MtPI/MtNGL9*, and Arabidopsis (L*er*) plants. We used the RNeasy Plant mini Kit (Qiagen) according to the manufacturer’s instructions. Total RNA was treated with rDNaseI of the DNase Treatment and Removal Kit (Ambion). For first-strand synthesis, total RNA (1 µg) was reverse transcribed in a 20 µl reaction mixture using the PrimerScript 1st strand cDNA Synthesis Kit (Takara). Aliquots of each cDNA were used as a template for real-time PCR or semi-quantitative RT–PCR with gene-specific primers (Supplementary Table S4). For real-time RT–PCR analysis, 1 μl of the reverse transcription reaction was used with 300nM of each primer mixed with the Power SYBR^®^ Green PCR Master Mix (Applied Biosystems) according to the manufacturer’s instructions. The reaction was carried out in 96-well optical reaction plates using an ABI PRISM 7500 Sequence Detection System and appropriate software (Applied Biosystems). The relative levels were determined by the 2^–ΔΔ*Ct*^ method. In all cases, the efficiency of the primers was analyzed. Each experiment was done with two biological replicates, each one with three technical replicates. To normalize the variance among samples, we used Secret Agent (*O*-linked *N*-acetyl glucosamine transferase: TC77416) (Hartweck *et al.*, 2002) for *M. truncatula*, *PsActin-11* (Zhang *et al.*, 2013) for *P. sativum* samples, elongation factor *MsEF-1* for *M. sativa*, and *Ubiquitin10* (UBQ10, At4g05320) (Czechowski *et al.*, 2005) for *A. thaliana*. All used primers are listed in Supplementary Table S4.

### Light microscopy and cryo-SEM

Images of *M. truncatula* R108, the *mtpi-2* and *mtngl9* mutant, Arabidopsis (L*er*) wild type, *35S::MtPI*, *35S::MtNGL9*, and the *pi-1* complementation lines flowers were obtained as described previously (Roque *et al.*, 2007). For cryo-scanning electron microscopy (SEM), samples were frozen in slush nitrogen and attached to the specimen holder of a CT-1000C cryo-transfer system interfaced with a JEOL JSM-5410 scanning electron microscope. The samples were then transferred from the cryostage to the microscope sample stage, where the condensed surface water was sublimed by controlled warming to –85 ºC. Afterwards, the sample was transferred back to the cryostage for gold coating by sputtering. Finally, the sample was returned to the microscope sample stage and viewed at an accelerating voltage of 15 keV.

### Analysis of adaptive evolution

We tested whether *MtPI* and *MtNGL9* gene copies diverged through adaptive changes in their protein-coding gene sequences after the duplication of their ancestral gene. To this end, we ran four maximum likelihood-based models, all of which are implemented in the program CODEML from the package PAML version 4.7 (Yang, 2007). These models are based on the calculation of the log-likelihood value of a particular model that supports a given hypothesis and that allows determination of the strength of selection on the gene by estimating the non-synonymous to synonymous rates ratio (ω=d_N_/d_S_). Values of ω=1, ω>1, and ω<1 indicate neutral evolution, positive selection, and purifying selection, respectively. For each of the models, we calculate a likelihood value and then compare the log-likelihood values among models by the likelihood ratio test (LRT), such that twice the difference in the likelihood values between two models under comparison can be approached to a χ^2^ distribution, with the degrees of freedom (df) being the number of parameters estimated by the most complex model and which are not estimated in the simplest model. We calculated the likelihood value for four models. The Goldman and Young model (Goldman and Young, 1994) assumes a single ω value for the entire tree and alignment. The free-ratio model (FRM) assumes a single ω for the alignment, but allows this value to be inferred for each branch of the tree, so that branches with adaptive evolution (ω>1) can be identified using this model. The branch site model (BSM) allows for ω values to change among tree branches and codon sites, and this model is compared with its null model in which ω is forced to be ω=1. The model that better fits the data and phylogeny is identified comparing the Goldman and Young model with the FRM, and the BSM with its null model.

### Analysis of functional divergence

We used two methods to identify functional divergence, one based on a maximum likelihood framework (Gu, 1999) and another based on a non-parametric approach (Caffrey *et al.*, 2012). The maximum likelihood approach tests for significant changes in the rates of evolution after gene duplication (Gu, 1999; Wang and Gu, 2001). To do so, this approach calculates the log-likelihood value under two hypotheses, one in which the parameter of functional divergence is larger than 0 (H_1_: θ > 0) and one with θ = 0 (H_0_). The log-likelihood values for both hypotheses are compared through an LRT (LRT = 2*Δ*l), with twice the difference in the log-likelihood values being approximated to a χ^2^ distribution with 1 df. If the null hypothesis is rejected, and thus we demonstrated the existence of functional divergence signatures at the sequence level, the posterior Bayesian probabilities at each of the amino acid sites in the alignment is calculated and those sites with probabilities >0.75 are assumed to have undergone changes responsible of the functional divergence between the two clades of paralogs. We tested two types of functional divergence, Type I and Type II. Type I functional divergence is given when at a particular amino acid site, the rate of evolution of one paralog is very slow while that of its sister clade is fast, indicating stronger functional constraints at that site in one paralog compared with the other, or a shift in the rates of evolution between the two paralogs (Gu, 1999). Alternatively, type II functional divergence measures a significant change in the amino acid properties at a site in the alignment between the two paralogous clades but with that site evolving at low rates in both of the paralogs (Gu, 2006). We used the software Diverge version 3.0 (Gu *et al.*, 2013) to test for functional divergence Types I and II. The non-parametric approach of Caffrey *et al.* (2012) uses a similar method to that of Gu but without specifying the phylogenetic clades under test, and thus allows identification of very strong signatures of functional divergence in particular branches of the phylogenetic tree. For each of the branches, the method estimates the parameter of functional divergence (FD) and tests whether this parameter is larger than that estimated from 1000 alignments that are simulated using the sequence and phylogenetic parameters of the real alignment.

## Results

### Expression pattern of *Mt PI*-like paralogs during floral development

**Fig. 1.**
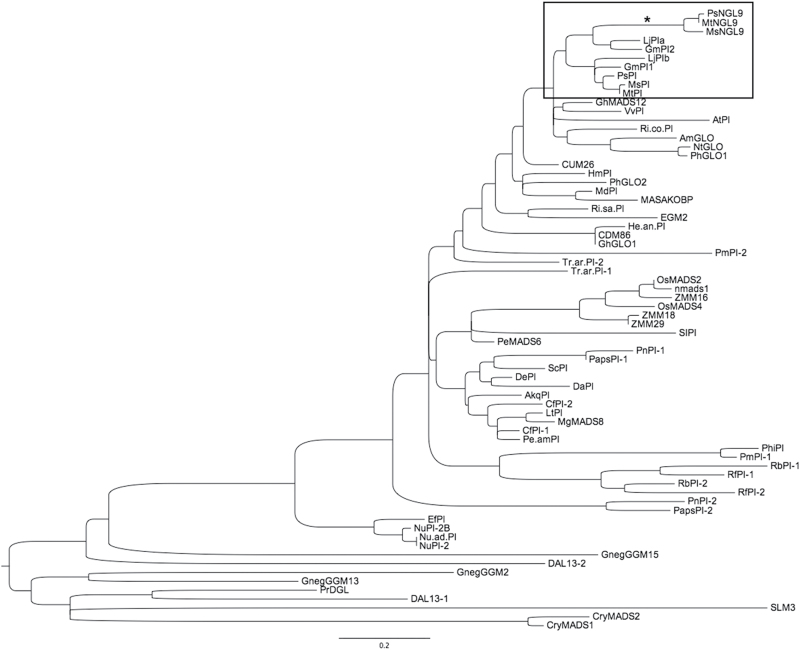
Phylogenetic tree of *PI*-like MADS-box genes. Sequences used in this analysis are listed in Supplementary Table S1. An accelerated branch incident on one of the *Medicago truncatula PI*-like gene paralogs is identified with an asterisk.

**Fig. 2.**
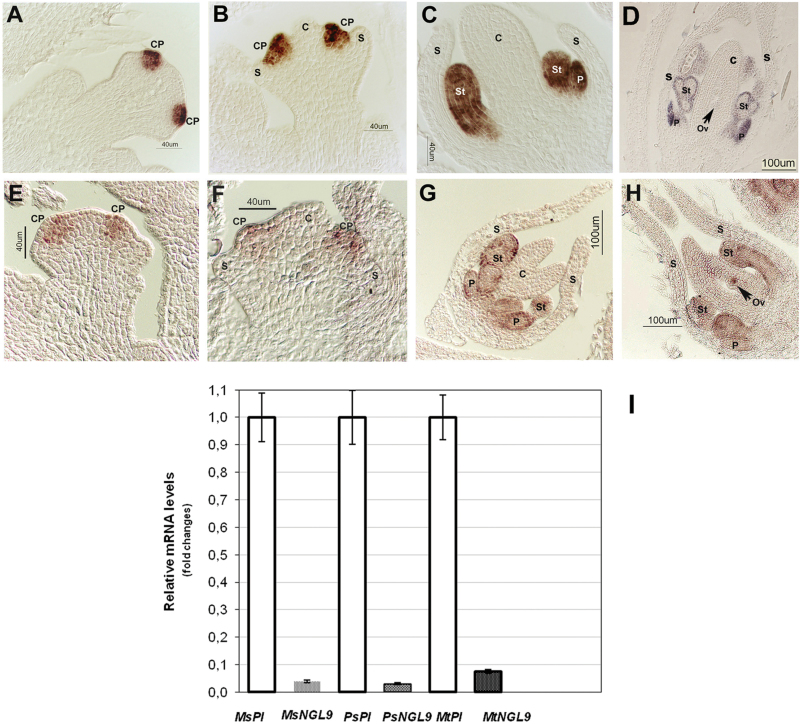
Expression patterns of the *M. truncatula PI*-like genes. *In situ* hybridization of the *MtPI* and *MtNGL9* mRNAs in *M. truncatula* wild-type flower buds. (A, B) *MtPI* expression was detected in cells of the common primordia (CP). (C, D) At late stages, *MtPI* was strongly expressed in the differentiated petals (P) and stamens (St). (E, F) *MtNGL9* was weakly expressed at stages 2 and 4 in cells of the common primordia. (G) At stage 5, *MtNGL9* mRNA was weakly detected in the cells of differentiated petals and stamens. (H) At stage 7, *MtNGL9* was also detected in ovules (Ov). (I) qRT–PCR analysis of *MtPI* and *MtNGL9* mRNA levels in floral buds of three leguminous specie*s.* The expression value of *MsPI*, *PsPI*, and *MtPI* genes was set to 1.00 and the expression levels of *MsNGL9*, *PsNGL9*, and *MtNGL9* were plotted relative to their respective *PI* values. (This figure is available in colour at *JXB* online.)

We previously isolated two *M. truncatula* B-function MADS-box genes (Benlloch *et al.*, 2009) belonging to the *PI/GLO* subfamily (*MtPI* and *MtNGL9*; Fig. 1), which are present in the *Medicago* genome as single copies (Supplementary Fig. S1). *MtPI* expression was detected at high levels in floral buds, whereas *MtNGL9* expression was very low (Fig. 2I). Similar differences in expression levels between the two *PI* paralogs were also observed in *P. sativum* and *M. sativa* floral buds (Fig. 2I). To determine if the *Mt PI*-like paralogs underwent spatial expression divergence during floral development, we compared the expression pattern of the two *MtPI* paralogs using *in situ* hybridization (Fig. 2A). *MtNGL9* mRNA was first detected in floral meristems at about stage 2, in the cells that give rise to common petal–stamen primordia (Benlloch *et al.*, 2003) (Fig. 2E). At stage 4, *MtNGL9* expression was observed only in the developing common primordia (Fig. 2F). *MtNGL9* expression was maintained during the entire process of common primordia compartmentalization and during the development of petals and stamens. (Fig. 2G, H). At late stages, *MtNGL9* mRNA expression was also detected in ovules (Fig. 2H). *MtPI* and *MtNGL9* had similar temporal and spatial expression patterns in petals and stamen during floral development (Fig. 2). While *MtPI* showed a higher uniformly distributed expression in petals and stamens during development, the *MtNGL9* signal seemed to be mainly confined to their epidermal cells in late developmental stages (Fig. 2G, H). Moreover, *MtNGL9* has a differential expression in ovules.

### Loss-of-function analyses of *MtNGL9* and *MtPI*

**Fig. 3.**
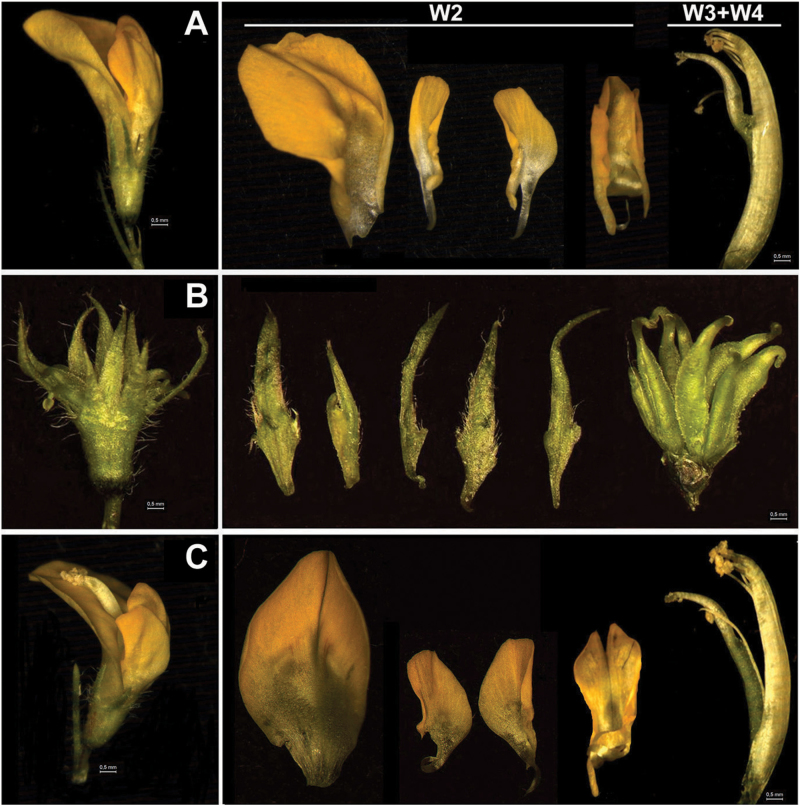
Phenotypes of *mtpi-2* and *mtngl9* mutants. (A) Wild-type *Medicago truncatula* flower. Petals present in the second whorl (W2), and stamens and carpel in W3+W4, respectively. (B) *mtpi-2* flower showing full conversion of petals into sepals and of stamens into carpels. (C) *mtngl9* flowers do not show homeotic floral alterations.

To investigate the specific contribution of the *MtNGL9* gene in *M. truncatula* floral development, we looked for retrotransposon insertion mutants (Cheng *et al.*, 2014). *mtngl9* mutants were isolated in a reverse genetics approach (see the Materials and methods). *mtngl9-1* contains a *Tnt1* insertion in the coding sequence 360bp from the start codon, in the second exon (Supplementary Fig. S2A) and *mtngl9-2* contains a *Tnt1* insertion in the coding sequence 183bp from the start codon, at the end of the first exon (Supplementary Fig. S2A). Homozygous plants were genotyped using specific primers for both insertion lines (Supplementary Fig. S2B, C). Gene expression analysis indicated that no *MtNGL9* transcript was detected in these plants (Supplementary Fig. S2D). We did not observe homeotic changes or any obvious mutant phenotype in floral organs (Fig. 3C). In contrast, *mtpi-2* plants (Cheng *et al.*, 2014) exhibited a complete conversion of petals to sepals and stamens to carpels (Fig. 3B). This phenotype resembles those of *MtPI*-RNAi flowers (Benlloch *et al.*, 2009). Knockdown of *MtPI* resulted in a marked reduction in the expression of all B-function MADS box genes (Supplementary Fig. S3A), while the loss of *MtNGL9* caused a slight decrease in their expression (Supplementary Fig. S3B). The full loss-of-B-function phenotype in *mtpi-2* and the pronounced effects of *MtPI* absence on the expression of the other B-function genes confirms *MtPI* as a master regulator in establishing the regulatory pathways for petal and stamen identity, while *MtNGL9* does not seem to have a role in this regard. Taken together, these results suggest that pseudogenization might be the functional evolutionary fate for this gene.

### Evolutionary analyses

To characterize the evolutionary forces acting on *MtPI*-like duplicated genes, we performed molecular evolution studies. Such studies rest on the assumption that functional genes are constrained by natural selection, while pseudogenes evolve under relaxed selective constraints (Petrov and Hartl, 2000; Zheng *et al.*, 2007; Tutar, 2012).

The phylogeny of *PI*-like genes reveals different rates of evolution between MtPI and MtNGL9 from different species (Fig. 1), with the lineage leading to the *MtNGL9* paralog (Fig.1, asterisk) being significantly longer (i.e. has a greater rate of amino acid evolution) than that leading to *MtPI* (Tajima’s relative rate test: χ^2^=5.45, df=1, *P*=0.019). This accelerated evolution in the branch leading to *MtNGL9* could be the result of relaxed selective constraints or be indicative of the positive selection of adaptive amino acid substitutions in that lineage.

To examine these two possibilities, we tested the action of positive selection in the *MtNGL9* lineage using the BSM as implemented in the program Codeml from the PAML package version 4.7 (Yang, 2007). Briefly, we compared the log-likelihood values for two hypotheses. The simplest hypothesis (i.e. null model or null hypothesis) assumes neutral evolution, hence relaxed constraints, in the branch of interest (the non-synonymous to synonymous rates ratio; ω=d_N_/d_S_=1). The alternative hypothesis, on the other hand, allows variable strengths of selection (ω) among codons in the lineage of interest (see the Material and methods). The value of ω is a good indicator of the strength of selection, with ω=1, ω<1, and ω>1 indicating neutral evolution, purifying selection, and positive selection, respectively. The model testing for adaptive evolution (BSM) detected several amino acid sites in the MADS-box domain under positive selection (ω 3.71; Supplementary Table S2), although this model did not improve the log-likelihood value of the null model [likelihood ratio test: simple model (BSMN) _0_= –10837.05, BSM _1_= –10836.37; χ^2^=1.36, 0.10<*P*<0.25). We also applied a model (the FRM) in which each branch of the tree was assumed to have a different ω value. This model showed, however, no evidence of positive selection in the branch leading to MtNGL9. The mean ω value for the entire tree and alignment, as estimated using the Goldman and Yang model (Goldman and Yang, 1994), was 0.14, indicative of strong purifying selection in this gene along the phylogenetic tree. The *MtNGL9* lineage showed an ω=0.56, supporting relaxed selective constraints, but no neutral evolution, in this branch. While these relaxed constraints could be indicative of a process of non-functionalization, the strong conserved amino acid nature of important functional sites in this gene and the fact that this value is lower than 1 indicate that this gene copy has evolved under strong purifying selection. Overall, these analyses discard pseudogenization (i.e. non-functionalization) as a plausible fate for the gene copy exhibiting an accelerated rate of evolution.

We also tested for amino acid substitutions that may have led to functional changes between the two genes generated by gene duplication. To this end, we examined the substitution patterns in each amino acid site of the multiple sequence alignment containing these genes. Sites that are functionally important but that have diverged after gene duplication should be highly conserved within each gene clade that includes ortholog genes from other species but variable between the paralogs. Inspection of these patterns identifies a number of sites that present a conserved amino acid in one gene copy which is different from its sister gene copy.

### No signatures of functional divergence between *MtNGL9* and *MtPI*

To test whether functional diversification events have followed the duplication giving rise to these two *Mt PI*-like gene copies, we searched for signatures of functional divergence in the amino acid sequences encoded by *MtNGL9* and *MtPI*. We used two tests of functional divergence: (i) a maximum-likelihood approach (see the Materials and methods) (Gu, 1999); and (ii) a non-parametric probabilistic approach (Caffrey *et al.*, 2012) that tests for functional divergence across the phylogenetic tree.

The maximum-likelihood test showed no evidence for either functional divergence Type I (parameter of functional divergence θ=0.087, *P*>0.1) or Type II (θ=0.016, *P*>0.1). Likewise, the non-parametric approach found no evidence of functional divergence within these groups (FD=0.01, *P*>0.25). Therefore, although some amino acid sites tended to be more conserved within paralogs than between them, formal statistical tests (Gu, 2003; Caffrey *et al.*, 2012; Gu *et al.*, 2013) could not find significant evidence supporting this observation.

### Interaction strengths between *Medicago truncatula* PI-like proteins

It has been shown that Arabidopsis PI and AP3 proteins act jointly as heterodimers to perform the B-function (Goto and Meyerowitz, 1994; Jack *et al.*, 1994; Riechmann *et al.*, 1996; Krizek and Meyerowitz, 1996; McGonigle *et al.*, 1996; Yang *et al.*, 2003). The heterodimer formed by PI- and AP3-like proteins is the only functional DNA-binding dimer in the vast majority of angiosperms (Melzer *et al.*, 2014).

**Fig. 4.**
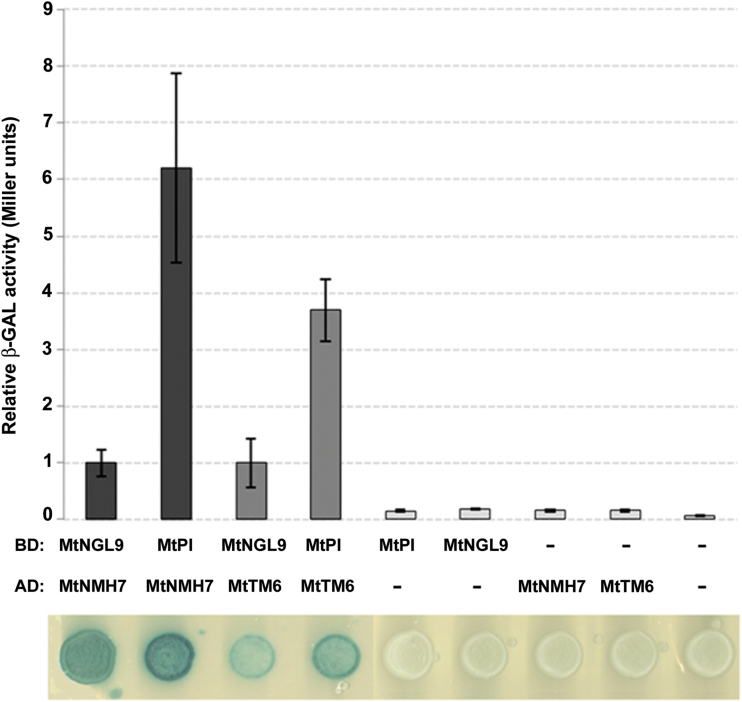
Quantification of the interaction between the *M. truncatula* B-function MADS-box proteins. BD, LexA-binding domain; AD, GAL4 activation domain. The β-GAL activity value of MtNGL9/MtNMH7 and MtNGL9/MtTM6 interactions was set to 1.00, and the MtPI/MtNMH7 and MtPI/MtTM6 interactions were plotted relative to these values, respectively. The values were calculated using 30 samples which correspond to 10 biological replicates. (This figure is available in colour at *JXB* online.)

 To evaluate the effect of relaxed functional constraints on the *MtNGL9* function, we carried out yeast two-hybrid assays on pairwise combinations of B-class MADS-box proteins to determine if the dimerization capabilities were conserved in *Mt PI*-like gene products. Our results showed that both MtPI and MtNGL9 were able to interact with the Mt AP3-like proteins MtNMH7 and MtTM6 (Fig. 4). Also, we analyzed whether differences exist in the strengths with which PI-type MADS-box proteins interact. We have quantified the interaction of Mt PI-like proteins with MtNMH7 and MtTM6 by β-galactosidase activity assays. The MtPI/MtNMH7 and MtPI/MtTM6 pairs produced stronger interactions as compared with the MtNGL9/MtNMH7 and MtNGL9/MtTM6 pairs (Fig. 4).

### Constitutive expression of *MtPI* and *MtNGL9* in Arabidopsis

**Fig. 5.**
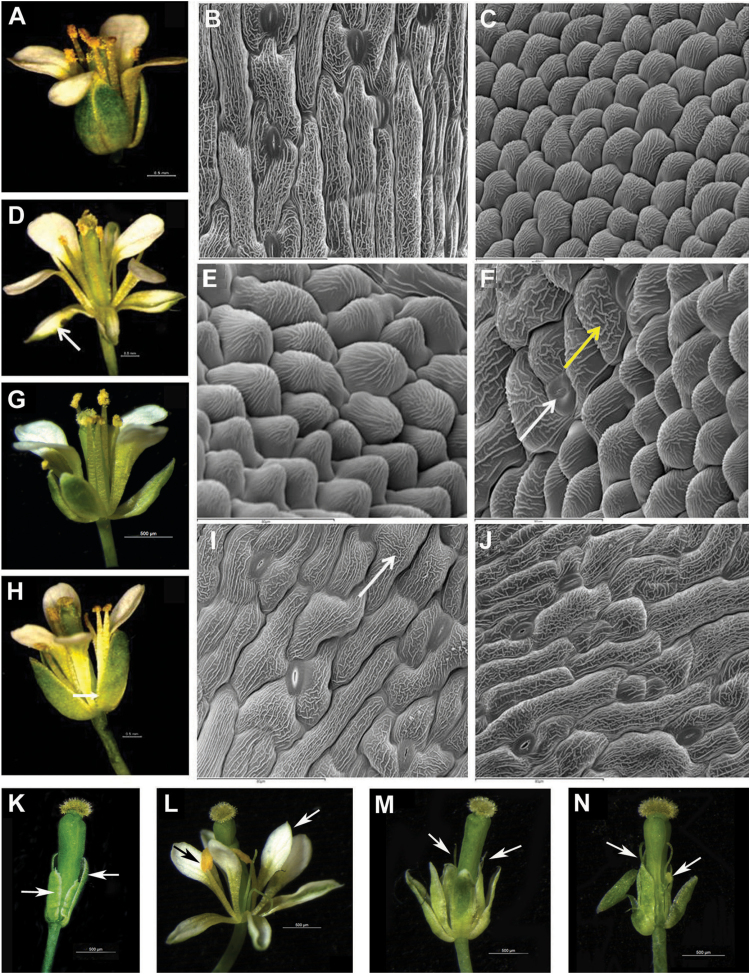
Constitutive expression of *MtPI* and *MtNGL9* in Arabidopsis and rescue of the *pi-1* mutant floral phenotype. (A) Wild-type flower (L*er*). (B) Abaxial epidermal cells of a wild-type sepal. (C) Abaxial epidermal cells of a wild-type petal. (D). Flower representative of the strong phenotype showing homeotic conversions of sepals into petals (arrow). (E, F) Abaxial epidermal cells of the first whorl organs of the *35S::MtPI* line displaying the strong phenotype. (G) Flower representative of the medium phenotype. (H) Flower representative of the weak phenotype. Arrow: slightly opened sepals. (I) Abaxial epidermal cells of the first whorl organs of a medium *35S::MtNGL9* transgenic line. (J) Abaxial epidermal cells of the first whorl organs of a weak *35S::MtNGL9* plant. (K) *pi-1* mutant flower. (L) Flowers from *35S*::*MtPI*;*pi-1* lines showing a near complete rescue of petal (white arrow) and stamen organs (black arrow). (M, N) *35S*::*MtNGL9*;*pi-1* flowers showing a weak rescue of petal and stamens (arrows).

To investigate the ability of MtPI and MtNGL9 proteins to induce B-function identity, we overexpressed these two genes in Arabidopsis plants. Constitutive expression of *MtPI* in Arabidopsis leads to homeotic conversion of sepals into petals (Fig. 5D). This phenotype is characteristic of the constitutive expression of most *PI*-like genes in Arabidopsis (Krizek and Meyerowitz, 1996; Berbel *et al.*, 2005). The majority of *35S*::*MtPI* (64.7%) plants displayed very open sepals transformed into petaloid organs (strong phenotype) (Fig. 5D). At the cellular level, these organs contained petal-like cells rounded and cobblestone-like in appearance with prominent cuticular thickenings (Fig. 5E). Occasionally, petal-like cells co-existed with cells retaining the typical pattern of the sepal cells, showing stomata (Fig. 5F, white arrow) and typical cuticular striation (Fig. 5F, yellow arrow). The remaining 35S::*MtPI* plants displayed flowers with partially opened sepals which occasionally showed a slight white color (medium phenotype) (Fig. 5G). In fact, the abaxial epidermal cells of the first whorl showed some cells with a small protrusion, which is reminiscent of cells typically found in petals (Fig. 5I; arrow). Most of the plants that overexpressed *MtNGL9* exhibited the medium phenotype (81.8%) described above (Supplementary Fig. S5C). The remaining *35S*::*MtNGL9* plants showed flowers with slightly opened sepals (weak phenotype; Fig. 5H) containing typical sepal-like cells (Fig. 5I).

We examined the expression levels of the *MtPI* and *MtNGL9* transgenes in their respective overexpression lines by qRT–PCR (Supplementary Fig. S5A, B). Despite the high expression levels of *MtNGL9* detected in *35S::MtNGL9* lines, neither of these lines displayed the strong phenotype (Fig. 5G–J). We were able to observe differences in the relative effects of both constructs, therefore indicating that the MtPI protein was more active than MtNGL9.

To determine if *Mt PI*-like genes could rescue petal and stamen organ identity in the absence of endogenous PI activity, we crossed heterozygous *35S::MtPI* and *35S::MtNGL9* transgenic lines into a *pi-1* mutant background. In the F_2_ population of kanamycin-resistant plants, a number of independent transformants homozygous for *pi-1* were identified (Supplementary Fig. S6), showing a range of phenotypes (Fig. 5L–N; Supplementary Table S3).

Only *pi-1/pi-1*;*35S::MtPI* plants showed near complete rescue of petal and stamen organs. Petals were indistinguishable from wild-type petals, and, in the third whorl, mosaic organs generally appeared, possessing both stamen and carpel characteristics. The restored stamens were not fully extended and did not produce fertile pollen (Fig. 5L; Supplementary Table S3). Meanwhile, *pi-1/pi-1*;*35S::MtNGL9* lines (Supplementary Table S3) showed a weak *pi-1* mutant rescue. The second whorl organs were larger than the second organs displayed in the *pi-1* mutant flowers with a whitish color, but they never reached the size of the wild-type petals (Fig. 5M, N). The third whorl organs were generally filament-like structures and occasionally the stamens showed anther-like organs (Fig. 5M, N). Similar levels of *MtPI* or *MtNGL9* transgenes were detected in floral buds of *pi-1/pi-1*;*35S::MtPI* or *pi-1/pi-1*;*35S::MtNGL9* lines, respectively (Supplementary Fig. S5), suggesting that the degree of rescue in the complementation lines is not correlated with the transgene expression level, but with the ability of MtPI or MtNGL9 to replace PI in *A. thaliana.*

## Discussion

### *MtPI* controls B-function in *Medicago truncatula*

Paralogous *Mt PI*-like genes arose from a duplication event that occurred prior to the speciation of legumes (Fig. 1). They may have originated during the whole-genome duplication (WGD) event that pre-dated speciation of *Mt* and other legumes ~50–60 Mya (Cannon *et al.*, 2006). Two copies of both *PI*-like genes are also found in the legume subfamilies *P. sativum*, *L. japonicus*, *M. sativa*, and *G. max*. Our phylogenetic analyses consistently placed one copy from each species in separate gene clades (Fig. 1). Our results showed that following this duplication event, only the *MtPI* duplicated copy has retained the B-function, based on its robust expression levels and the conserved expression pattern of the B-class genes, protein interaction capabilities, and the strong homeotic phenotype observed for the *mtpi-2* mutant. The co-ordinated expression of *MtPI* with *MtNMH7* in the inner cell layers of the petal and stamen organ primordia and with *MtTM6* in the outer primordia cell layers is required to specify petal and stamen identity in *M. truncatula* (Roque *et al.*, 2013). However, *MtNGL9* does not appear to have a role in this regard, so could potentially be on the way to becoming a pseudogene.

### *MtNGL9* does not show evidence of pseudogenization

Duplicate genes that are stably preserved in genomes usually have divergent functions (He and Zhang, 2005). Indeed, duplicated genes destined to ‘die’ usually do so within a few million years after duplication, having acquired mutations that result in a non-functional gene (Lynch and Conery, 2000). Pseudogenes are expected to evolve neutrally; therefore, after a sufficient amount of time, the signature of purifying selection at the amino acid level will ultimately be erased (Zou *et al.*, 2009).

*mtngl9* mutants do not present any homeotic change or any obvious floral mutant phenotype (Fig. 3C), suggesting that *MtNGL9* does not have a role in the specification of petal and stamen fate. Our analyses discard pseudogenization as a plausible scenario for *MtNGL9* evolution. First, the rate of evolution of *MtNGL9* indicates strong purifying selection (i.e. dN/dS <1). Secondly, *MtNGL9* has persisted for >60 million years, as the duplication of the gene pre-dates the speciation of legumes (Cannon *et al.*, 2006). Since the half-life for a duplicated gene is in the order of 20 million years (Lynch, 2007), then we can assume that *MtNGL9* has not lost its function.

In the basal eudicot *Papaver somniferum*, one of the *Paps* PI-like proteins has lost dimerization capabilities with the *Paps* AP3-like proteins, thus indicating that this gene could be on the way to becoming a pseudogene in this species (Drea *et al.*, 2007). In contrast, in support of a functional role for *MtNGL9*, this gene encodes a protein that conserves this competence to interact with the Mt AP3-like proteins, which it is an essential feature for the B-class MADS box proteins.

### Mode of *Medicago truncatula PISTILLATA*-like gene evolution after duplication

Studies of MIKC^C^-type MADS-box TFs in an array of species are contributing to a better understanding of how these genes may have changed their functions after duplication (Vandenbussche *et al.*, 2004; Drea *et al.*, 2007; Geuten and Irish, 2010; Pan *et al.*, 2010; Fourquin *et al.*, 2013; Roque *et al.*, 2013; Serwatowska *et al.*, 2014).

A number of theoretical models have been proposed to explain the persistence of duplicated genes in genomes. Paralogs may be selected for increased dosage or as a repository for gene conversion against deleterious changes in either copy and result in functional redundancy (Nadeau and Sankoff, 1997; Nowak *et al.*, 1997; Gu, 2003; Gu *et al.*, 2003). Alternatively, the paralogs may diverge either to generate new gene functions (neofunctionalization) (Taylor and Raes, 2004) or to partition multiple functions (subfunctionalization) through complementary degeneration (Force *et al.*, 1999; Stoltzfus, 1999; Lynch and Force, 2000). These theoretical expectations are, however, only partially consistent with data (Lynch and Conery, 2000).

To describe the evolution of these duplicated genes, we have to invoke factors from different models of molecular evolution. The differential evolution of the two clades examined here is in agreement with the quantitative subfunctionalization model of Force and colleagues (Force *et al.*, 1999): the unduplicated ancestor possessed elevated expression levels that were retained in only an *Mt PI*-like gene copy after the WGD pre-dating legumes speciation, the *MtPI* gene. Similar differences in expression levels between the two *PI* paralogs were also observed in *P. sativum* and *M. sativa* floral apices (Fig. 2I), suggesting ancestral changes in regulatory sequences. How can this differential expression between the two gene copies explain their evolutionary and functional patterns?

Whether gene expression can evolve independently from gene function remains an open question in evolutionary biology (Castillo-Davis *et al.*, 2004). Many recent studies have explored molecular and population-genetic constraints on the rate of protein evolution and they have hypothesized that the best predictor of the evolutionary rates of proteins is the gene expression level (Pal *et al.*, 2001; Herbeck *et al.*, 2003; Rocha and Danchin, 2004; Subramanian and Kumar, 2004; Gout *et al.*, 2010). Our results showed that the expression levels may have imposed additional constraints to the highly expressed MtPI protein. The lower expression of the *MtNGL9* gene is likely to have relaxed the selective constraints on this gene, in agreement with the mistranslation model (Drummond *et al.*, 2005), allowing it to evolve at a faster rate than *MtPI*. *MtNGL9* has lost its ancestral expression level, and, as a consequence, its ancestral function seems to have decreased. In line with this, the constitutive expression of *MtPI* and *MtNGL9* in Arabidopsis and the rescue of the *pi-1* mutant by these genes revealed that MtPI had a greater ability than MtNGL9 to be a component of the transcriptional complex that specifies the second and the third whorl in a heterologous system.

TFs that function in the context of molecular networks are likely to be under stronger selective pressures (Luisi *et al.*, 2015). Thus, the most parsimonious explanation for the preservation of both *Mt PI*-like duplicated gene copies is that both will be selected to achieve an optimal total dosage balance of their functional complex. In the case of ohnologs (duplicated genes generated by WGD), expression dosage is thought frequently to drive functional persistence, suggesting that the stoichiometry of expression among duplicated loci is important (Papp *et al.*, 2003; Birchler and Veitia, 2007; Edger and Pires, 2009; Gout *et al.*, 2010; Makino and McLysaght, 2012). *MtNGL9* would have a far lower contribution to the total activity required to specify the B-function. The slight decrease of *MtPI* and the other B-class gene expression levels in *mtngl9* floral buds suggests that *MtNGL9* may be required to maintain the critical dosage for the B-function in *M. truncatula*.

On the other hand, we have detected *MtNGL9* expression in ovules (Fig. 2H). The B-class gene *MtNMH7* (Roque *et al.*, 2013) and the euAP3 gene *GmNMH7* in soybean (Wu *et al.*, 2006) have also been detected in this floral tissue. Moreover, low levels of *MtNGL9* transcript have been reported in vegetative tissues (Benlloch *et al.*, 2009). The presence of transcripts in a given tissue does not necessarily mean that the gene activity is required for its proper development. However, it might be considered that changes in *MtNGL9* regulation enabled its expression in different tissues and that the preservation of *MtNGL9* and its partial functional relaxation subsequently provide the opportunity for the fixation of advantageous mutations, which can lead to new functions (He and Zhang, 2005). This makes neofunctionalization plausible as an alternative evolutionary fate for this gene, through the ability to form part of new complexes that control other development processes. The extent to which such divergences have led to a neofunctionalization of one of the duplicated *MtPI*-like copies and possible acquisition of other roles in different developmental processes requires further investigation.

Our results provide evidence of the complex dynamics underlying the evolution by gene duplication of the B-class MADS-box subfamily. Notwithstanding this complexity, the B-function remains conserved in all eudicots, providing robustness to the specification of petal and stamen identities. Finally, our findings confirm MADS-box TFs as promising targets for novel studies aimed at a more complete understanding of the complexity of evolution by gene duplication.

## Supplementary data

Supplementary data are available at *JXB* online.

Figure S1. Southern blot analysis of the *Medicago truncatula PI*-like genes.

Figure S2. Molecular characterization of the *Tnt1* insertions in the *MtNGL9* locus.

Figure S3. qRT–PCR expression analyses of loss-of-function plants.

Figure S4. Analyses of *35S:MtPI* and *35S:MtNGL9* plants.

Figure S5. RT–PCR expression analyses in *pi-1/pi-1*;*35S:MtPI* and *pi-1/pi-1*;*35S:MtNGL9* complementation lines.

Figure S6. Analyses of the *pi-1* complementation by *MtPI* and *MtNGL9.*

Table S1. Sequences from different plant species used in the elaboration of the phylogenetic tree with their respective GenBank accession numbers.

Table S2. Maximum-likelihood test of adaptive evolution in the duplicated *M. truncatula PISTILATA*-like transcription factors.

Table S3. Summary of rescue phenotypes.

Table S4. Primers used in this work.

Supplementary Data
