# Supplementary material for: Evolution by gene duplication of Medicago truncatula PISTILLATA-like transcription factors
Source: J Exp Bot. 2016 Jan 15;67(6):1805–17. doi: 10.1093/jxb/erv571 (PMC4783364; doi:10.1093/jxb/erv571)
Supplement: Supplementary Data [file supp_67_6_1805__index.html]

Evolution by gene duplication of Medicago truncatula PISTILLATA-like transcription factors — Evolution by gene duplication of Medicago truncatula PISTILLATA-like transcription factors — Supplementary Data 

# Evolution by gene duplication of *Medicago truncatula PISTILLATA*-like transcription factors

## Supplementary Data

Data files

- supplementary\_figures\_S1\_S6\_tables\_S1\_S4.pdf - Supplementary Data
