# Supplementary material for: Evolution by gene duplication of Medicago truncatula PISTILLATA-like transcription factors
Source: J Exp Bot. 2016 Jan 15;67(6):1805–17. doi: 10.1093/jxb/erv571 (PMC4783364; doi:10.1093/jxb/erv571)
Supplement: Supplementary Data [file supp_erv571_supplementary_figures_S1_S6_tables_S1_S4.pdf]

Evolution by gene duplication of *Medicago truncatula PISTILLATA*-like transcription factors. Roque E. *et al.*

SUPPLEMENTARY DATA

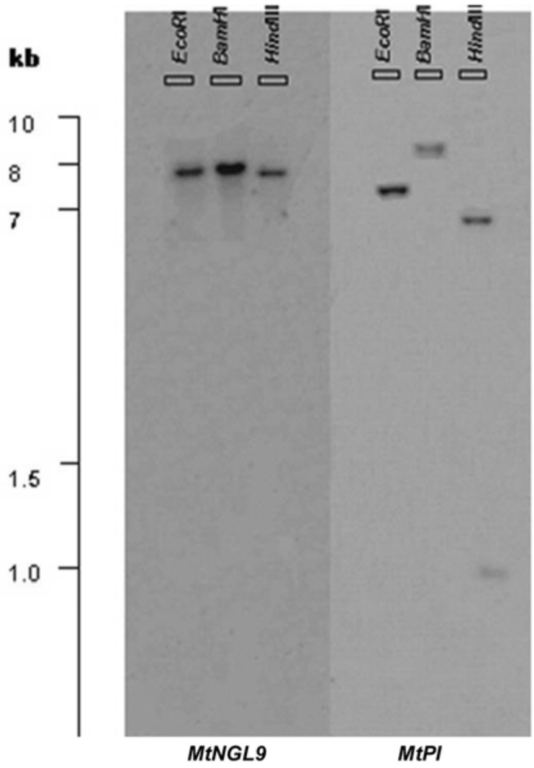

Fig. S1. Southern blot analysis of the *Medicago truncatula PI*-like genes. *MtPI* and *MtNGL9* are present in the *Medicago* genome as single copies.

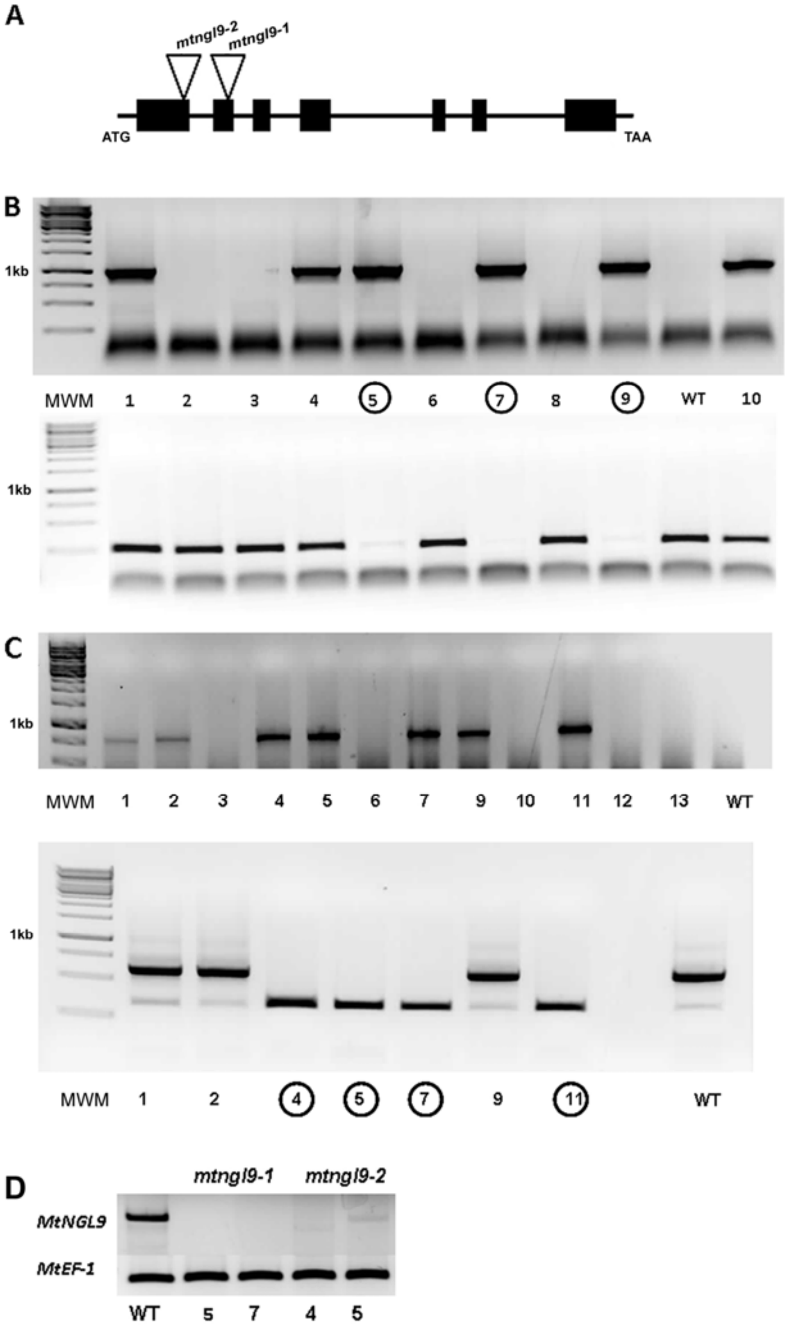

Fig. S2. Molecular characterization of the *Tnt1* insertions in the *MtNGL9* locus. A. Schematic diagram of the genomic structure of the *MtNGL9* gene. In NF14948 lines (*mtngl9-1*), *Tnt1* was inserted 360 bp downstream of the ATG codon, at the second exon. In NF12316 lines (*mtngl9-2*). *Tnt1* was inserted 183 bp downstream of the ATG codon, at the end of the first exon. **B.** Genotyping of *mtngl9-1* mutant using NGL9-F/Tnt1-R (upper) and NGL9-291/NGL9-612G (lower) primer pairs. Homozygous plants are indicated in circled numbers. **C.** Genotyping of *mtngl9-2* mutant using NGL9-F/Tnt1-R (upper) and NGL9-F/NGL9-590G (lower) primer pairs. Homozygous plants are indicated in circled numbers. **D.** RT-PCR results show no detectable *MtNGL9* expression in four homozygous mutant plants. MWM: molecular weight markers; WT: wild-type control.

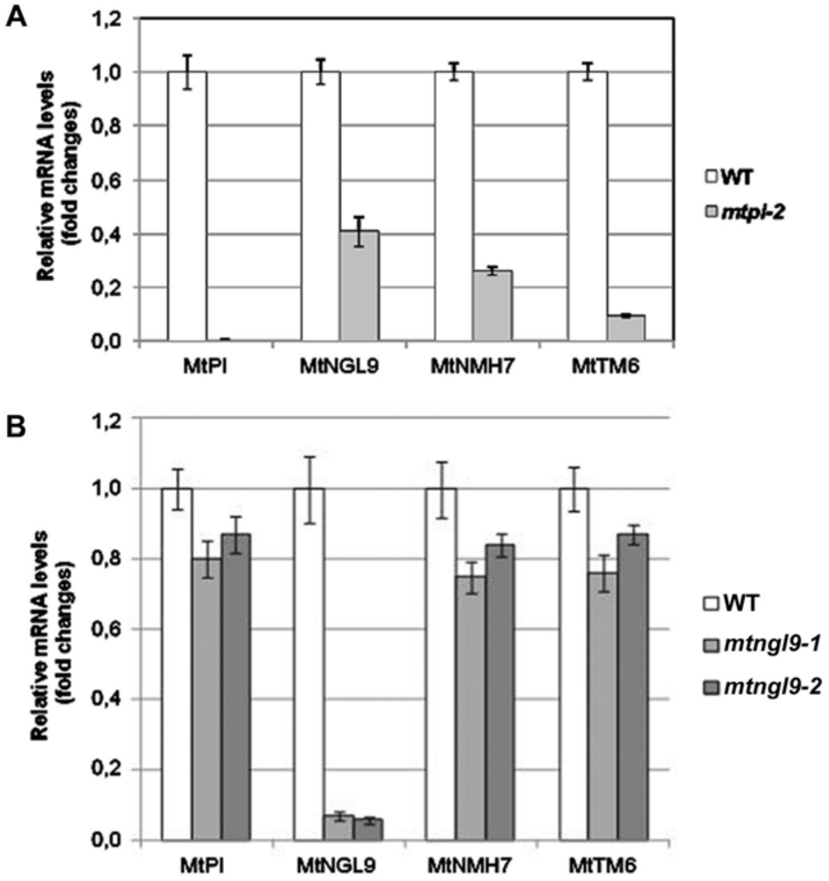

Fig. S3. qRT-PCR expression analyses of loss-of-function plants. A. mRNA levels of the B-function genes in floral buds of *mtpi-2.* **B.** mRNA levels of the B-function genes in floral buds of *mtngl9-1* and *mtngl9-2*. The height of the bars for a given gene indicates relative differences in expression levels in floral buds. The expression value of all B-function genes in WT was set to 1.00. Expression levels were plotted relative to this value.

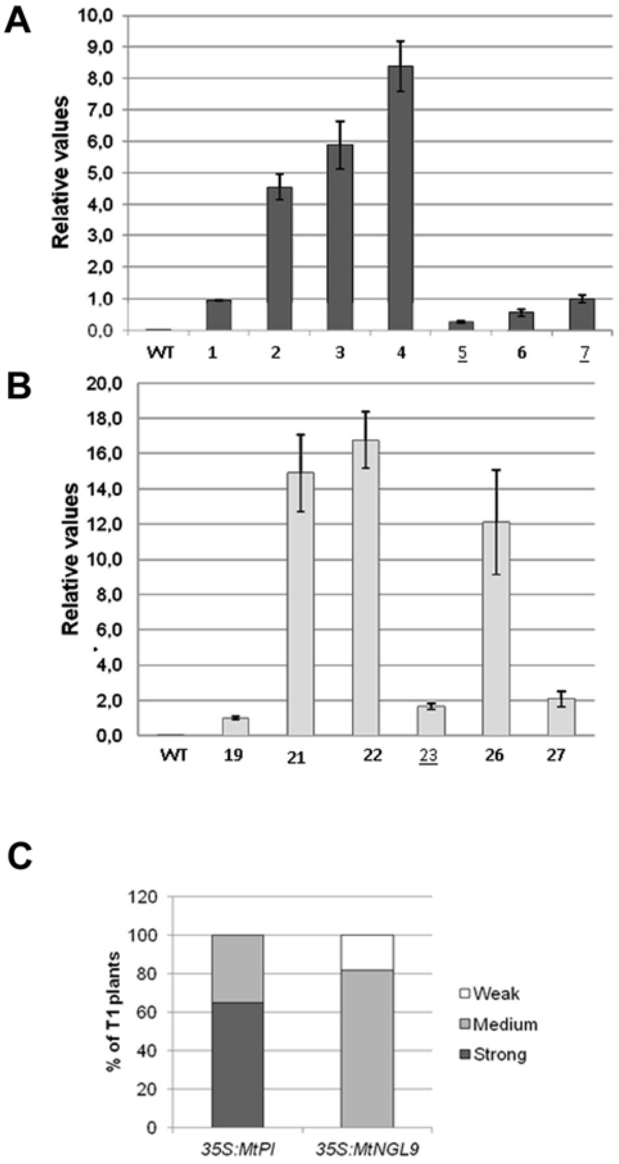

Fig. S4. Analyses of *35S:MtPI* and *35S:MtNGL9* plants. A. Transcript abundance of *MtPI* in 35S*::MtPI* plants. The expression value of *MtPI* in plant number 7 was set to 1.00. Expression levels were plotted relative to this value. Numbers indicate the transgenic line. Underlined number indicates plants classified as medium phenotype, and the remaining plants showed the strong phenotype. **B.** Transcript abundance of *MtNGL9* in 35S*::MtNGL9* plants. The expression value of *MtPI* in plant number 19 was set to 1.00. The number of the transgenic line is indicated. Underlined number indicates plants classified as weak phenotype, and the remaining plants showed the medium phenotype. **C.** Relative proportion of phenotypes observed in plants overexpressing *MtPI* (N^{o} of transgenic plants= 17) or *MtNGL9* (N^{o} of transgenic plants= 33).

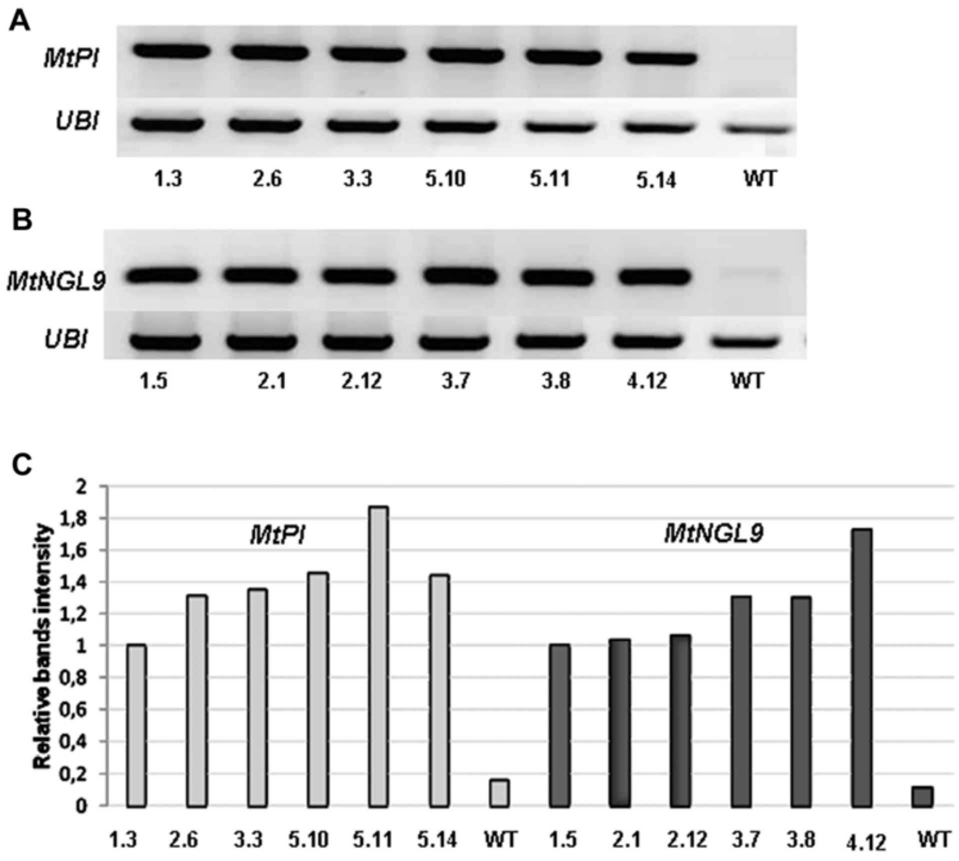

Fig. S5. RT-PCR expression analyses in *pi-1/pi-1*; *35S:MtPI* and *pi-1/pi-1*; *35S:MtNGL9* complementation lines. A. Expression of *MtPI* in *pi-1/pi-1*; *35S:MtPI* lines. **B.** Expression of *MtNGL9* in *pi-1/pi-1*; *35S:MtNGL9 lines.* **C.** Comparative expression level of *MtPI* and *MtNGL9* in their respective complementation lines. Levels were quantified by measuring the intensity of individual bands using ImageJ densitometry software, and expressed relative to UBIQUITIN signal, as a measure of transcript relative abundance in the different samples. The numbers of the transgenic lines are indicated.

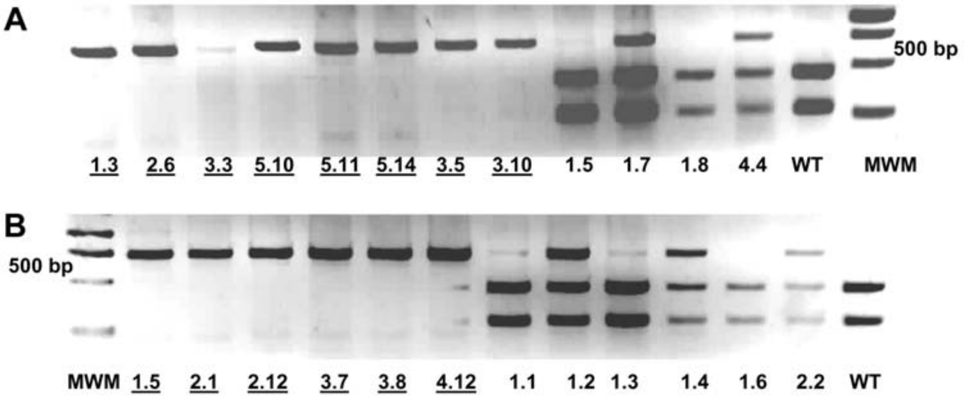

Fig. S6. Analyses of the *pi-1* complementation by *MtPI* and *MtNGL9.* A. Genotyping of some individuals of the complementation lines by *MtPI.* **B.** Genotyping of some individuals of the complementation lines by *MtNGL9***.** Genomic PCR product (729 bp) was digested by *Bse*GI. *pi-1*/*PI*; *35::MtPI* or *pi-1*/*PI*; *35S::MtNGL9* background resulting in three fragments: 729, 451 and 278 bp. Two fragments (278 and 451 bp) were obtained in the case of wild-type plants (*PI*/*PI*; *35S::MtPI* or *PI*/*PI*; *35S::MtNGL9*. *Bse*GI does not cut the 729 bp PCR fragment in *pi-1*/*pi-1; 35::MtPI* or *pi-1*/*pi-1; 35::MtNGL9* background (underlined numbers).

Classification	Taxa	PI-homologues	Gymnosperm B-class genes
**GYMNOSPERMS**			
Coniferales	*Picea abies*		*DAL13-1* (AF158539)
Pinaceae			*DAL13-2* (AF158541)
	Pinus radiata		PrDGL (AF120097)
Cupressaceae	*Cryptomeria japonica*		*CryMADS2* (AF097747)
			CryMADS1 (AF097746)
Gnetaceae	*Gnetum gnemon*		*GnegGGM2* (AJ132201)
			GnegGGM13 (AJ132219)
			GnegGGM15 (AJ251555)
**ANGIOSPERMS**			
Nymphaceae	*Nuphar japonica*	*Nj PI-1* (AB158359)	
		NjPI-2 (AB158360)	
	Nuphar advena	*Nu.ad.PI* (AY337736)	
	Euryale ferox	*EfPI* (AB158350)	
MAGNOLIIDS			
Magnoliaceae	*Liriodendrum tulipifera*	*LtPI* (AF052864)	
	Magnolia praecocissima		
		MpMADS8 (AB050650)	
Laurales			
Calycanthaceae	*Calycanthus floridus*	*CfPI-1* (AF230708)	
		CfPI-2 (AF230709)	
Lauraceae	*Persea americana*	*Pe.am.PI* (AY337738)	
Piperales			
Piperaceae	*Peperomia hirta*	*PhiPI* (AF052865)	
	Piper magnificum	*PmPI-1* (AF052866)	
		PmPI-2 (AF052867)	
**MONOCOTS**			
Poales			
Poaceae	*Oryza sativa*	*OsMADS2* (L37526)	
		OsMADS4 (L37527)	
		nmads1 (AF095645)	
	Zea mays	*ZMM16* (AJ292959)	
		ZMM18 (AJ292960)	
		ZMM29 (AJ292961)	
Liliales			
Liliaceae			
	Phalaenopsis equestris	*PeMADS6* (AY678299)	
Alismatales			
Alismataceae	*Sagittaria montevidensis*	*SmPI* (AF230712)	
**EUDICOTS** (early divergent eudicots)			
Ranunculales	*Papaver nadicaule*	*PnPI-1* (AF052855)	
		PnPI-2 (AF052856)	
Papaveraceae	*Papaver somniferum*	*PapsPI-1* (EF071994)	
		PapspI-2 (EF071995)	
	Sanguinaria canadiensis	*ScPI* (AF130871)	
	Akebia kinata	*AkqPI* (AY162837)	
	Ranunculus bulbosus	*RbPI-1* (AF052859)	
		RbPI-2 (AF052860)	
	Ranunculus ficaria		
Ranunculaceae		*RfPI-1* (AF052858)	
	Delphinum ajacis	*RfPI-2* (AF130872)	
	Trochodendron aralioides	DaPI (AF052862)	
Trochodendraceae		*Tr.ar. PI-1* (EF436259)	
		Tr.ar. PI-2 (EF436260)	
**CORE EUDICOTS**			
Caryophyllales			
Caryophyllaceae	*Silene latifolia*	*SLM3* (X89108)	
Saxifragales			
Saxifragaceae	*Ribes sanguineum*	*Ri.sa. PI* (AY337742)	
**ROSIDS**			
Euforbiáceas			
Euphorbiaceae	*Ricinus communis*	*Ri.co. PI* (XM_002520262)	
Vitales		*VvPI* (DQ059750.1)	
Vitaceae	*Vitis Vinifera*		

Myrtales		*EGM2* (AF029976)	
Myrtaceae	*Eucalyptus grandis*		
Eurosid I			
Cucurbitales		*CUM26* (AF043255)	
Cucurbitaceae	*Cucumis sativus*		
Fabales		*MsNGL9* (AF335473)	
Fabaceae	*Medicago sativa*	***MsPI*** (KJ470631)	
		MtPI (FJ403468)	
	Medicago trunctula	*MtNGL9* (FJ403469)	
		PsPI (AY842791)	
	Pisum sativum	***PsNGL9*** (KJ470632)	
		LjPIa (AY770398)	
	Lotus japonicus	*LjPIb* (AY770399)	
		GmPI1 (BT089901)	
	Glycine max	*GmPI2* (BT090476)	
Malvales			
Malvaceae			
		GhMADS12 (FJ409869)	
Rosales	*Gossypium hirsutum*		
Rosaceae			
		MdPI (AJ291490)	
EurosidsII	*Malus domestica*	MASAKO BP (AB038462)	
	Rosa rugosa		
		AtPI (D30807)	
Brassicales			
Brassicaceae	*Arabidopsis Thaliana*		
**ASTERIDS**			
Cornales			
Hydrangeaceae	*Hydrangea macrophylla*		
		HmPI (AF230711)	
EuasteridsI			
	Antirrhinum majus		
Lamiales			
Plantaginaceae		*AmGLO* (X68831)	
	Nicotiana tabacum		
Solanales	*Petunia hybrida*		
Solanaceae		*NtGLO* (X67959)	
		PhGLO1 (M91190)	
		PhGLO2 (X69947)	
Euasterids II		*GGLO1*(AJ009726)	
	Gerbera hybrida	*CDM86* (AY173061)	
Asterales	*Chrysanthemun morifolium*		
Asteraceae	*Helliantus annuus*	*He.an.PI* (AY157725)	

Table S1. Sequences from different plant species used in the elaboration of the phylogenetic tree with their respective GenBank accession numbers.

Model	Log-likelihood (ℓ)	Parameter estimates	Amino acids
G&Y	-10937.758	ω = 0.147	-
FRM	-10773.329	-	-
BSMN	-10837.050	ω_0 = 0.144, P_0= 0.79; ω_1 = 1, P_1= 0.21	-
BSM	-10836.367	ω_0 = 0.144, P_0= 0.68; ω_1 = 1, P_1= 0.18, ω_2 = 3.71, P_2= 0.11	Leu9, Ser47, Ser14, Arg40, Ala74.

Table S2. Maximum-likelihood test of adaptive evolution in the duplicated *M. truncatula PISTILATA*-like transcription factors. To identify signatures of adaptive evolution after gene duplication, we conducted four models, all implemented in the program PAML package version 4.7 (Yang, 2007): (a) G&Y model estimates the non-synonymous-to-synonymous rates ratio (ω) for the entire alignment and phylogenetic tree, (b) FRM model (Free-ratio model), estimates one ω per phylogenetic branch, (c) BSMN refers to the neutral branch-site model and allows inferring ω under a neutral evolution model (0< ω≤1) for a user-specified branch of the tree, which in this case was the branch leading to the MtNGL9 duplicate, and (d) BSM model is the branch-site model that allows ω to be greater than 1, hence making it possible the identification of positive selection. The proportion of sites under each ω category (Pi) was also estimated.

Genotype	Weak rescue	Strong rescue	N^{o}.of F2 kanamycin resistant plants	N^{o}.of expected *pi-1/pi-1* plants
pi-1/ pi-1*; *35S::MtPI	5 (26,3 %)	14 (73,7 %)	65	16,24 (1/4)
pi-1/ pi-1; 35S::MtNGL9	14 (100%)	0	64	16 (1/4)

Table S3. Summary of rescue phenotypes.

Primer name	Sequence 5'-3'	Use
**MtNGL9-ATG**	ATGGGAAGAGGAAAGATTGAG	For *PsNGL9* cDNA isolation
**MtNGL9-638**	GCTGAAGTAGTGAGTGAAGC	
**MtPI-ATG**	ATGGGAAGAGGTAAGATTGAG	For *MsPI* cDNA isolation
**MtPI-548**	GGTTAAATCCATTGTCCATGCA	
**MsPI-363**	GATGGCCCTTGAGGAATCCCTT	
**MtNGL9-511**	GGCAGTGGAAGGTGGGAGATAA	For southern blot hybridization and RNA *in situ* hybridization
**MtNGL9-753**	GAAAGTGTAAGCCCAGGGATC	
**MtPI-504**	GGCACTTGAAGGTGTGGGAAA	
**MtPI-801**	GAAACCAAATTCAATCACTTCATA	
**MtPI-SalI**	GGTCGACCCATGGGAAGAGGTAAGATTGAG	For overexpression constructs
**MtPI-BamHI**	GCGGATCCTTAAATCCATTGTCCATGCATG	
**MtNGL9-SalI**	GGTCGACCCATGGGAAGAGGAAAGATTGAG	
**MtNGL9-BamHI**	CGGGATCCTTATAATCCATTTATCTCCCA	
**BAD-MtPI-FOR**	GCTGGATCCGCATGGGAAGAGGTAAGATTGAG	For yeast two-hybrid constructs
**BD-MtPI-REV**	GTCGTCGACTTAAATCCATTGTCCATGCATG	
**BAD-MtNGL9-FOR**	GCTGGATCCGCATGGGAAGAGGAAAGATTGAG	
**BD-MtNGL9-REV**	GTCGTCGACAAAGCTGAAGTAGTGAGTGAAG	
**IKC-MtNMH7-FOR**	GCTGGATCCGCGCCTCAACAAAGCAGTTTTTCGAT	
**AD-MtNMH7-REV**	GTCGAATTCCTAAAAAAGTAAAGGGTAAGTGGTG	
**IKC-MtTM6-FOR**	GCTGGATCCGCCTCTCTACAAAGAAGATTATTGATC	
**AD-MtTM6-REV**	GTCCTCGAGTCAACCCAGGCGCAAATCGTCA	
**Tnt1-R**	CAGTGAACGAGCAGAACCTGTG	For *mtngl9* mutants genotyping
**NGL9-F**	GGGAAGAGGAAAGATTGAGATC	
**NGL9-590G**	GCTACCTGAGCTGAATTTGCATGTC	
**NGL9-612G**	CCAACCATATATAGCTAATCTAGCTACCTG	
**CAPS-FOR**	CCTAAAATTGAGAAAGAGATAGAGAG	For *pi-1* mutant genotyping
**CAPS-REV**	CGCATAAGATTTGGTCTTGATGGTTTC	
**MtPI qRT-FOR**	GCTGATGGCCCTTGAGGAAT	For qRT-PCR
**MtPI qRT-REV**	TCACGGACGCCAGTAAGACC	
**MtNGL9 qRT-FOR**	AACTCAAGAAAGAGAATGAGGA	
**MtNGL9 qRT-REV**	TAAGACATCCTCTAAGCTCATC	
**MtNMH7 qRT-FOR**	TTAGGTCCACGCATGTTTGC	
**MtNMH7 qRT-REV**	AGGATTAGGATGAGTAGGCTGTAAGC	
**MtTM6 qRT-FOR**	CGAAGGAGACGAGGAATCAG	
**MtTM6 qRT-REV**	AGAGTGGAAGCACCATTGGC	
**Sec.Ag-qRT-FOR**	TGGCTACTAGGGTTGCTGGC	
**Sec.Ag-qRT-REV**	CCTCACCCAGTCCAGTGGAA	
**MsNGL9 qRT-FOR**	ATAAAGAAGCTGAATCTGGGTTTTTG	
**MsNGL9 qRT-REV**	ACACGCATATATAATGTAATGTAACCTCAA	
**MsPI qRT-FOR**	GTGTGTTTCCTAAATAGCCATTGATC	
**MsPI qRT-REV**	GCCATATTAAAGCAACATACATTTTTTC	
**PsPI qRT-FOR**	TGGACAATGGATTTAACCAGATCA	
**PsPI qRT-REV**	GCATAATATTGAGCAAACTAATTCCCT	
**PsNGL9 qRT-FOR**	TGATGAATTTGATGAGAGTACTGGAAG	
**PsNGL9 qRT-REV**	GAAAATGGCATCTGGGAATTGA	
**PsACTIN-11-FOR**	AGGTGCTGTACCAACCATCCA	
**PsACTIN-11-REV**	CGTGAATTCCTGCTGCTTCC	
**MsNGL9 qRT-FOR**	ATAAAGAAGCTGAATCTGGGTTTTTG	
**MsNGL9 qRT-REV**	ACACGCATATATAATGTAATGTAACCTCAA	
**MsPI qRT-FOR**	GTGTGTTTCCTAAATAGCCATTGATC	
**MsPI qRT-REV**	GCCATATTAAAGCAACATACATTTTTTC	
**MsEF-1-FOR**	CAGGCTGATTGTGCAGTCCTT	
**MsEF-1-REV**	CCAGCTTCAAAACCACCAGTG	
**AtUBQ FOR**	GGCCTTGTATAATCCCTGATGAATAAG	
**AtUBQ REV**	AAAGAGATAACAGGAACGGAAACATA	
**MtNGL9 qRT-FOR**	GCTGATGGCCCTTGAGGAAT	For semi-quantitative RT-PCR
**MtNGL9-507 REV**	CCCTTGTTGCTGCTGGAGGATA	
**MtPI-363**	GATGGCCCTTGAGGAATCCCTT	
**MtPI-548**	GGTTAAATCCATTGTCCATGCA	
**MtEF-1-FOR**	AGAAGGAAGCTGCTGAGATGAAC	
**MtEF-1-REV**	CTGCTTCACTCCAAGAGTAAATGC	
**AtUBQ FOR-S**	GATCTTTGCCGAAAACAATTGGAGGATGGT	
**AtUBQ REV-S**	CGACTTGTCATTAGAAAGAAAGAGATAACAGG	

Table S4. Primers used in this work.
